# Perceived Neighborhood Racial Composition and Multimorbidity Among Young, Middle-Aged, and Older Black Americans

**DOI:** 10.1093/geroni/igaf122.1824

**Published:** 2025-12-31

**Authors:** Corina Mills, Courtney Thomas Tobin, Angela Gutierrez, Aisha Fletcher, Reeya Patel, James Huynh, Roland Thorpe

**Affiliations:** Johns Hopkins Bloomberg School of Public Health, Baltimore, Maryland, United States; University of California, Los Angeles Fielding School of Public Health, Los Angeles, California, United States; Western University of Health Sciences, Pomona, California, United States; University of California, Los Angeles, Los Angeles, California, United States; University of California, Los Angeles, Los Angeles, California, United States; University of Michigan, Ann Arbor, Michigan, United States; Johns Hopkins Bloomberg School of Public Health, Baltimore, Maryland, United States

## Abstract

Multimorbidity, the co-occurrence of two or more chronic health conditions, is a major public health challenge in the U.S., disproportionately affecting Black Americans. Neighborhood conditions are crucial determinants of health disparities, affecting healthcare access, exposure to stressors, and availability of resources for disease management. However, the impact of individuals’ perceptions about the residents of their community on multimorbidity remains underexplored. This study examined the association between perceived racial neighborhood composition (PNRC) and multimorbidity among young (18-35 years), middle-aged (36-49 years), and older (50 – 69 years) Black Americans. Data were drawn from 627 Black adults in the Nashville Stress and Health Study. Multimorbidity was based on the sum of 11 chronic physical health conditions. PNRC was derived from participants’ assessment of the relative proportions of Black and White people in their neighborhoods [(0) Mostly Black, (1) About Half Black, (1) Mostly White]. Age-group-stratified negative binomial regression models were estimated, adjusting for gender, SES, and marital status. Results indicate that young adults who perceived living in half Black neighborhoods had more chronic conditions [incident rate ratio (IRR) = 1.81, 95% confidence interval (CI): 1.01-3.25] than young adults who perceived living in mostly Black neighborhoods. Older adults who perceived living in mostly White neighborhoods reported fewer chronic conditions [IRR = 0.77, 95% CI: 0.61-0.97] than older adults living in mostly Black neighborhoods. Findings demonstrate the distinct impact of PNRC across age groups, underscoring the need for age-specific, place-based interventions to address multimorbidity among Black Americans.

